# Supplementary material for: V2a interneuron diversity tailors spinal circuit organization to control the vigor of locomotor movements
Source: Nat Commun. 2018 Aug 22;9:3370. doi: 10.1038/s41467-018-05827-9 (PMC6105610; doi:10.1038/s41467-018-05827-9)
Supplement: Supplementary file 1 — Supplementary Information [file 41467_2018_5827_MOESM1_ESM.pdf]

SUPPLEMENTARY INFORMATION

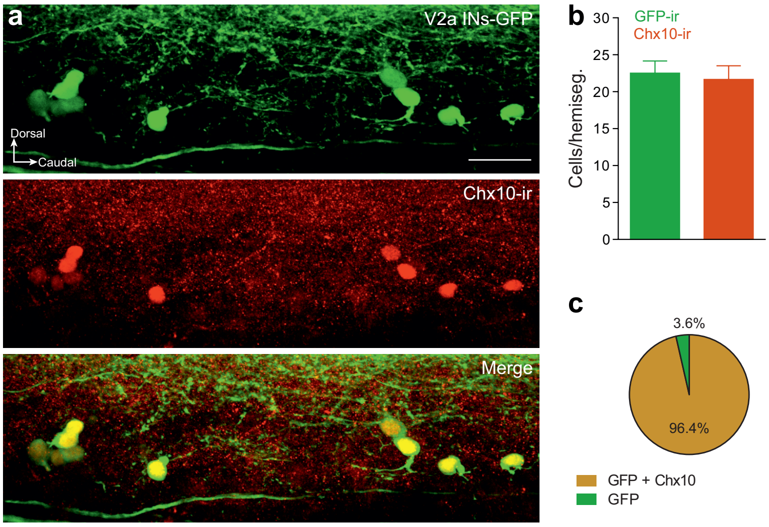

Supplementary Fig. 1. Co-localization of GFP and *Chx10* in V2a interneurons. (a) Side view of the spinal cord showing that GFP-expressing V2a interneurons are *Chx10*-immuonoreactive. **(b)** Number of neurons per hemi-segment expressing GFP and *Chx10*-ir (n = 3 animals; error bars in the graph reflect the s.e.m.). **(c)** Fraction of neurons co-localizing GFP and *Chx10*. Scale bar, 20 µm

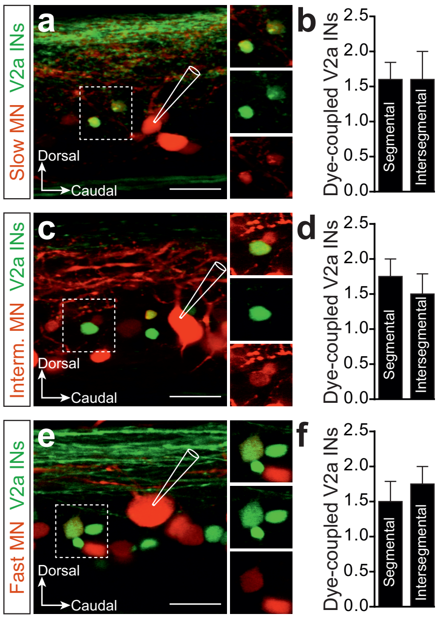

Supplementary Fig. 2. Convergence of V2a interneurons onto single motoneurons. (a) Intracellular injection of neurobiotin into a slow motoneuron resulted in dye-coupling in V2a interneurons. **(b)** Number of segmental and intersegmental V2a interneurons dye-coupled to single slow motoneurons (*n* = 5 motoneurons from separate animals, error bars reflect the s.e.m.). **(c)** V2a interneurons dye-coupled to an intermediate motoneuron. **(d)** Number of segmental and intersegmental V2a interneurons dye-coupled to single intermediate motoneurons (*n* = 5 motoneurons from separate animals, error bars in the graph reflect the s.e.m.). **(e)** V2a interneurons dye-coupled to a fast motoneuron. **(f)** Number V2a interneurons converging onto single fast motoneurons (*n* = 5 motoneurons from separate animals, error bars in the graph reflect the s.e.m.). Scale bars, 20 µm

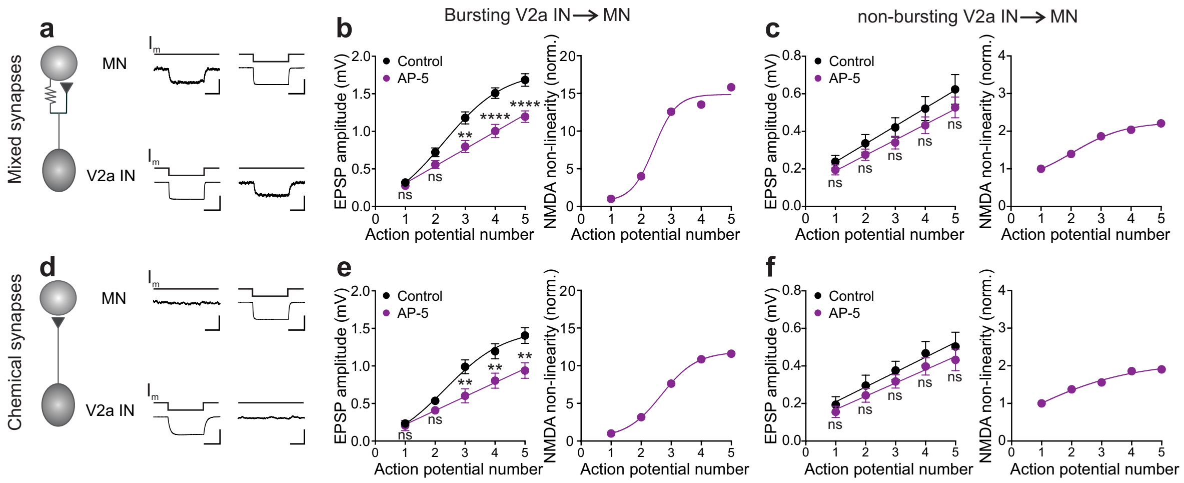

Supplementary Fig. 3. Contribution of NMDA receptors in connections with mixed synapses and only chemical synapses. (a-c) In pairs of V2a interneurons and motoneurons connected with mixed electrical and chemical synapses, blockade of NMDA receptors with AP-5 (100 µM) affected the non-linear summation induced by bursting-type V2a interneurons **(b)** (interaction: $F_{4,50}$ = 4.487, P = 0.0043; two-way repeated-measures ANOVA; n = 6; ****P<0.0001; **P<0.01; error bars in the graph reflect the s.e.m.), but not the linear summation induced by non-bursting-type V2a interneurons **(c)** (interaction: $F_{4,50}$ = 0.095, P = 0.9834; two-way repeated-measures ANOVA; n = 6; error bars in the graph reflect the s.e.m.). **(d-f)** In pairs of V2a interneurons and motoneurons connected only with chemical synapses, AP-5 affected the non-linear summation induced by bursting-type V2a interneurons **(e)** (interaction: $F_{4,50}$ = 2.65, P = 0.0439; two-way repeated-measures ANOVA; n = 6; **P<0.01; error bars in the graph reflect the s.e.m.), but not the linear summation induced by non-bursting-type V2a interneurons **(f)** (interaction: $F_{4,50}$ = 0.038, P = 0.9972; two-way repeated-measures ANOVA; n = 6; error bars in the graph reflect the s.e.m.). Scale bars, 100 ms, 20 mV, 0.5 mV

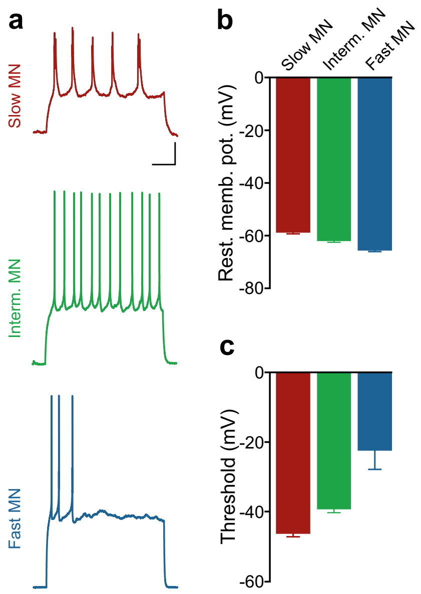

Supplementary Fig. 4. Properties of motoneurons of the three modules. (a) Firing pattern of motoneurons of the slow, intermediate and fast modules. **(b)** Resting membrane potential of slow, intermediate and fast motoneurons ($n = 10$ of each motoneuron type; error bars in the graph reflect the s.e.m.). **(c)** Threshold for action potentials in slow, intermediate and slow motoneurons ($n = 10$ of each motoneuron types; error bars in the graph reflect the s.e.m.). Scale bars, 100 ms, 10 mV
